# Increased levels of reactive oxygen species in platelets and platelet-derived microparticles and the risk of respiratory failure in HIV/AIDS patients

**DOI:** 10.1590/0074-02760200082

**Published:** 2020-09-14

**Authors:** Wellington Mota Gama, Lucas Barbosa Oliveira, Yury Oliveira Chaves, Flavio Ribeiro, Taynná Vernalha Rocha Almeida, Barbara Jose Antunes Baptista, Monique Freire Santana, Luis Carlos Ferreira, Marcus Vinicius Guimaraes Lacerda, Paulo Afonso Nogueira

**Affiliations:** 1 Universidade Federal do Amazonas, Programa de Pós-Graduação em Imunologia Básica e Aplicada, Manaus, AM, Brasil; 2 Fundação Oswaldo Cruz-Fiocruz, Instituto Leônidas e Maria Deane, Programa de Pós-Graduação em Biologia da Relação Patógeno-Hospedeiro, Manaus, AM, Brasil; 3 Fundação Oswaldo Cruz-Fiocruz, Instituto Oswaldo Cruz, Programa de Pós-Graduação em Biologia Parasitária, Rio de Janeiro, RJ, Brasil; 4 Fundação de Medicina Tropical Heitor Vieira Dourado, Instituto de Pesquisa Clínica Carlos Borborema, Manaus, AM, Brasil; 5 Universidade Federal do Amazonas, Programa de Pós-Graduação em Ciências da Saúde, Manaus, AM, Brasil; 6 Universidade do Estado do Amazonas, Programa de Pós-Graduação em Medicina Tropical, Manaus, AM, Brasil

**Keywords:** HIV/AIDS, respiratory failure, reactive oxygen species, platelets and platelet-derived microparticles

## Abstract

Respiratory failure (RF) is the main cause of hospital admission in HIV/AIDS patients. This study assessed comorbidities and laboratory parameters in HIV/AIDS inpatients with RF (N = 58) in relation to those without RF (N = 36). Tuberculosis showed a huge relative risk and platelet counts were slightly higher in HIV/AIDS inpatients with RF. A flow cytometry assay for reactive oxygen species (ROS) showed lower levels in platelets of these patients in relation to the healthy subjects. However, when stimulated with adrenaline, ROS levels increased in platelets and platelet-derived microparticles of HIV/AIDS inpatients, which may increase the risk of RF during HIV and tuberculosis (HIV-TB) coinfection.

Respiratory diseases of infectious origin are the most commonly occurring and principal causes of death in HIV/AIDS patients.^1^ ^,^ ^2^ Lethality rates are notoriously high in patients co-infected with HIV and tuberculosis (HIV-TB), since the virus weakens the host’s immune response to *Mycobacterium tuberculosis*, thus the chances of developing a TB infection are greatly increased and its progression is more dramatic.^2^ ^,^ ^3^

The respiratory conditions in these patients can be aggravated by the platelet activation process.^4^ In HIV-1 infections, platelets have been shown to circulate in an activated state and their degree has been correlated with the severity of disease.^5^ However, this persistent state of activation in these patients may cause them to become them refractory to further stimulation. One study assessed *ex vivo* platelet function in HIV-infected subjects and showed hyper- and hypo-reactivity in platelets depending on the platelet agonist tested. While platelet aggregation is increased in response to adrenaline, collagen and thrombin-receptor agonist peptides induced significantly less aggregation.^6^ Another showed a depressed release of the chemokine RANTES, which indicates a deficit in platelet function in HIV patients.^7^ Despite increased levels of platelet activators and exacerbation of platelet activation, it is known that HIV infection is often associated with a deficit in platelet function or a state of “platelet exhaustion”.^7^

This ambiguity has been shown to have a relationship with the lungs, since on the one hand platelets contribute as a pulmonary vascular barrier and in defense against pulmonary hemorrhage, though on the other hand, they contribute to pathologic syndromes of pulmonary inflammation and thrombosis.^4^ Recently, we observed a relationship between increased PDW and likelihood of survival in HIV/AIDS inpatients. Opportunistic infections were the major events in mortality of these patients, which evidences the role of antimicrobial host defense under severe immunosuppression (Gama WM et al., Unpublished observations).

The lack of data on platelet involvement in HIV infection is mainly associated with respiratory comorbidities and requires further investigation. Here, we evaluated the hematological and biochemical data of ninety-four patients hospitalised with HIV/AIDS, who were admitted to the Fundação de Medicina Tropical - Dr Heitor Vieira Dourado (FMT-HVD) in the Amazonas State, Brazil. It was a prospective study and sampling was done for convenience. Patient engagement in the study was carried out with the patient or his/her companion, according to ethical procedures (CAAE: 57330116.6.0000.0005) and information regarding comorbidities were obtained from the electronic medical database at the FMT-HVD after consent was obtained. Patients were classified into two groups, those with RF (n = 58) and those (n = 36) with no RF (Table).

**TABLE:** Comparison of comorbidities and clinical data of HIV/AIDS patients with or without respiratory failure

Comorbidities	RF	No RF	RR	95%CI	p
	n (%)	n (%)			
Gender	44(75.8)	27(75)	1.011	0.8025 to 1.325	0.9999
HAART	46(79.3)	24(66.7)	1.19	0.9302 to 1.61	0.2247
Death	16(27.5)	5(13.9)	1.986	0.8473 to 4.931	0.1361
TB	38(65.5)	1(2.7)	23.59	4.571 to 134	<0.0001
Neurological syndromes	25(43.1)	15(41.6)	1.034	0.6475 to 1.716	0.9999
Cardiovascular comorbidities	2(3.4)	3(8.3)	0.4138	0.0858 to 1.998	0.3676
Digestive syndromes	14(24.1)	10(27.7)	0.869	0.4431 to 1.751	0.8086
Weight loss	29(50)	18(50)	1	0.6691 to 1.549	0.9999
Diarrhea	18(31)	11(30.5)	1.016	0.5571 to 1.918	0.9999
Vomiting	18(31)	17(47.2)	0.6572	0.3942 to 1.108	0.1296
Clinical parameters^*^	Median (IQ25;IQ75)	Median (IQ25;IQ75)			p
Age	37(28.75;43)	34.5(29;41.75)			0.6111
HIV-RNA copies/mL	35397(397;173958)	34884(180;498626)			0.5577
CD4-T cells/µL (600 to 1;500/mm3)	60(18.75;234)	84(28;169.8)			0.9826
CD8-T cells/µL (200 to 800/mm3)	544.5(338;1124)	519(292;1041)			0.8519
CD4/CD8 ratio (1.0 and 4.0)	0.15(0.06;0.405)	0.15(0.0575;0.3025)			0.5353
Hemoglobin, g/dL (13.0-18.0 g/dL)	10.5(8765;12.89)	9.7(8.16;12.29)			0.1360
Leukocytes/mL (4;500-11;000/mL)	4180(3290;7200)	4995(3888;7405)			0.3191
Lymphocyte (%)	23.01(17;36)	24(12.75;34.05)			0.8209
Neutrophils/mL (1,800-7,700/mL)	2756(1711;4347)	3552(2542;5021)			0.0543
Monocytes/mL (80-1;100/mL)	295(148.8;418.5)	395.5(209.8;519.8)			0.1493
Eosinophils/mL (40-550/mL)	3(2;6.4)	3.6(2;9.75)			0.5924
Platelets ×10^3^/mL (150;000-4000;000×10^3^/mL)	297000(209750;415000)	263000(123500;319750)			0.0344
MPV (8.8 fL a 12.5 fL)	8.06(7.36;8.96)	8.32(7825;8.58)			0.3543
PDW ( 9.3 fL a 16.0 fL)	13.5(11.63;16.75)	14.13(12.25;17.38)			0.4495
Bilirubin, mg/dL (<1.0 mg/dL)	0.39(0.28;0.73)	0.59(0.36;1.55)			0.0270
Creatinine, mg/dL (0.6-1.35 mg/dL)	0.8(0.6;1)	0.8(0.6;1.2)			0.5041
DHL, U/uL (120 e 246 U/uL)	384.5(306.5;487.3)	343(285.8;485.3)			0.3875
Gamma-glutamyltransferase, U/uL^**^	91(58.75;217)	89.5(42.25;413.8)			0.7336
Albumin, g/dL (3.5-5.0g/dL)	3.9(3.35;4.5)	3.7(2.59;4.1)			0.1044
Alkaline phosphatase, U/uL (65.0-330.0 U/uL)	287(202.5;403)	296(204;496)			0.8218
Aspartate aminotransferase, U/uL (2-38.0U/uL)	32(25;59)	39(25.25;60.75)			0.4931
Alanine aminotransferase, U/uL (2-44.0U/uL)	41(23.5;74.5)	37(23.25;80.25)			0.7640

*: for some parameters, parenthesis indicate normal range values; **: references values of Gamma-glutamyltransferase (men: 10 to 50 U/uL and women: 7 to 32 U/uL).

RF was classified as a respiratory syndrome when patients reported dyspnea (shortness of breath or difficulty in breathing), atypical chest performance with abnormal noises, long-term or productive cough, vesicular breathing sounds, abnormal respiratory murmurs, pleural effusion, gasping and wheezing. Pulmonary coinfections were tuberculosis, pneumocystosis and pulmonary histoplasmosis. Participants considered as having tuberculosis were those with positive Xpert MTB/RIF test results and positive sputum smears for *M. tuberculosis*. During hospitalisation, all HIV-TB coinfected inpatients underwent the 4-drug regimen anti-TB treatment (isoniazid, rifamycin, pyrazinamide and ethambutol).

Other comorbidities were defined as signs and/or symptoms of neurological and digestive origin, of infectious and non-infectious origin, with or without chronicity. Neurological syndromes were classified as disorientation, seizure, paralysis, movement deficit, mental confusion, tumor, hemiplegia, depression and mental disorder, psychotic depression, organic mental disorder and dementia. Neurological coinfections were neurocriptococcosis, neurotoxoplasmosis, neurotuberculosis, and meningoencephalitis. Circulatory syndromes were hypertension, endocarditis, pericardial tuberculosis, heart failure, megaloblastic anemia, edemigenic syndrome and hematological syndrome. Digestive syndromes were classified as erosive esophagitis, ulcerative esophagitis, digestive hemorrhage, gastritis and epigastria, abdominal pain, odynophagia, oroscopy, intestinal lymphadenomegaly, nephrotoxicity, gastritis and diarrheal syndrome. Most digestive syndromes were oral candidiasis, moniliasis with whitish lesions, esophageal candidiasis, intestinal tuberculosis, amoebic colitis and anal condyloma. Other digestive comorbidities, such as chronic lymphocytic leukemia, Hodgkin’s disease, aplastic anemia, and neurological disorders e.g., multiple sclerosis and myasthenia gravis, were monitored.

The probability of TB or neurological, circulatory, and digestive syndromes that are a comorbidity of HIV inpatients with RF was assessed using the Chi Square test (Table). TB showed a twenty-three-fold higher risk among patients with RF, and represented the major cause of hospitalisation. Logistic regression predicted TB as major cause of respiratory syndrome [crude Odds Ratio (OR) = 0.02, 95% confidence interval (CI) = 0.0-0.14) and adjusted OR = 0.01 95% CI = 0.0-0.08) P-likelihood-ratio test < 0.001], was as observed in other studies.^8^ ^,^ ^9^ Patients with vomiting were 58% less likely to have RF [crude OR = 0.42 (95% CI = 0.17-1.02) and adj. OR = 0.17 (95% CI = 0.04-0.7) P-likelihood-ratio test = 0.007]. In addition, hematological and biochemical parameters were collected from the electronic medical record closest to the interview day. Despite having statistical differences, bilirubin levels were in the normal range (Table). On the other hand, levels of hepatic and cardiac serum enzymes were higher in both groups, and indicated chronicity of HIV infection.

Of all the evaluated markers, the T-test showed that the mean of platelet counts was slightly higher in patients with RF (Table). This slight increase in platelet counts of patients with RF may be due to the fact that reactive thrombocytosis is linked to pulmonary tuberculosis, as observed in other studies.^8^ ^,^ ^9^ ^,^ ^10^ ^,^ ^11^ The clinical situations in HIV patients with respiratory diseases can be further aggravated by the platelet activation process, which is induced by the exacerbation of the constant inflammatory state in these patients. Platelets are best known as primary mediators of hemostasis and may be targets for reactive oxygen species (ROS) during cell activation.^12^ ROSs are known to modulate the coagulation and fibrinolysis pathways in platelet activation, and promote coagulation initiation and activation of other coagulation factors.^12^ An imbalance between the production and detoxification of these ROSs may drastically affect platelet physiology and even lead to, as a final event, a change in the number of cells. The dihydroethidium (DHE) is a fluorochrome that allows the characterisation of redox responses in platelet activation by physiological and pathological stimuli.^13^ For ROS analysis in platelets and microparticles, for convenience, a small group of samples (N = 21) taken within 72 h of admission from the patients hospitalised with HIV was investigated. Fifty-two percent of inpatients (n = 11) had RF; 42.9% were HIV-TB coinfected patients, 95.2% reported effective use of HAART; 9.5% died, 52.4% had a neurological comorbidity, 19.0% cardiovascular disorder and 38.1% presented a digestive comorbidity. The age average of this small group of HIV inpatients was 38.0 ± 10.3 years and the level of ROS was compared in paired-age samples collected from twenty-three healthy volunteers. The blood collection was performed after signing of the informed consent form.

The platelet-rich plasma (PRP) was obtained with two cycles of centrifugation at 500 x g for 20 min at 25ºC in PSG buffer (5 mM of PIPES, 145 mM of NaCl, 4 mM of KCl, 50 μM of Na2HPO4, 1 mM of MgCl_2_•6H_2_O, 5.5 mM of glucose; pH 6.8 and 300 nM of Prostaglandin E1, according to previous studies,^14^ the platelets were re-suspended in this buffer and labeled with anti CD41 and DHE for platelet marking and visualisation using a FACSCanto™ II cytometer (BD Biosciences, USA). The Flowjo Software was used to select platelet population by Forward versus Side Scatter (FSC vs SSC; Figure). The total number of double platelets (labeled CD41+DHE+) was calculated from the acquisition of 10,000 events (Figure A). The total number of CD41 + DHE + platelets did not differ from those of healthy people (Figure B). PRPs were stimulated with 200 µg/mL of adrenaline (Hipolabor 1mg/mL) for 30 min and then labeled, fixed and acquired on the cytometer. In relation to the level of ROS before stimulation, HIV platelets showed lower levels than the control ones. However, after adrenaline stimulation, the level of ROS in HIV platelets measured by mean of fluorescence intensity (MFI) increased when compared to control platelets (Figure C). The low ROS level before stimulation may suggest platelet exhaustion in HIV infection, as observed by Holme and colleagues with time-dependent platelet aggregation.^7^ Meanwhile, Satchell and colleagues showed differences in reactivity of platelets to agonist in HIV-infected patients possibly mediated through effects at both receptor and post-receptor levels.^6^ There are few studies on ROS in human platelets,^13^ and analysis of ROS generation in HIV patients is new. In this study, platelets of HIV patients stimulated by adrenaline were more reactive due to the increase of MFI. Therefore, our research underlines the need for further prospective studies regarding platelet function in HIV patients.

**Figure:**
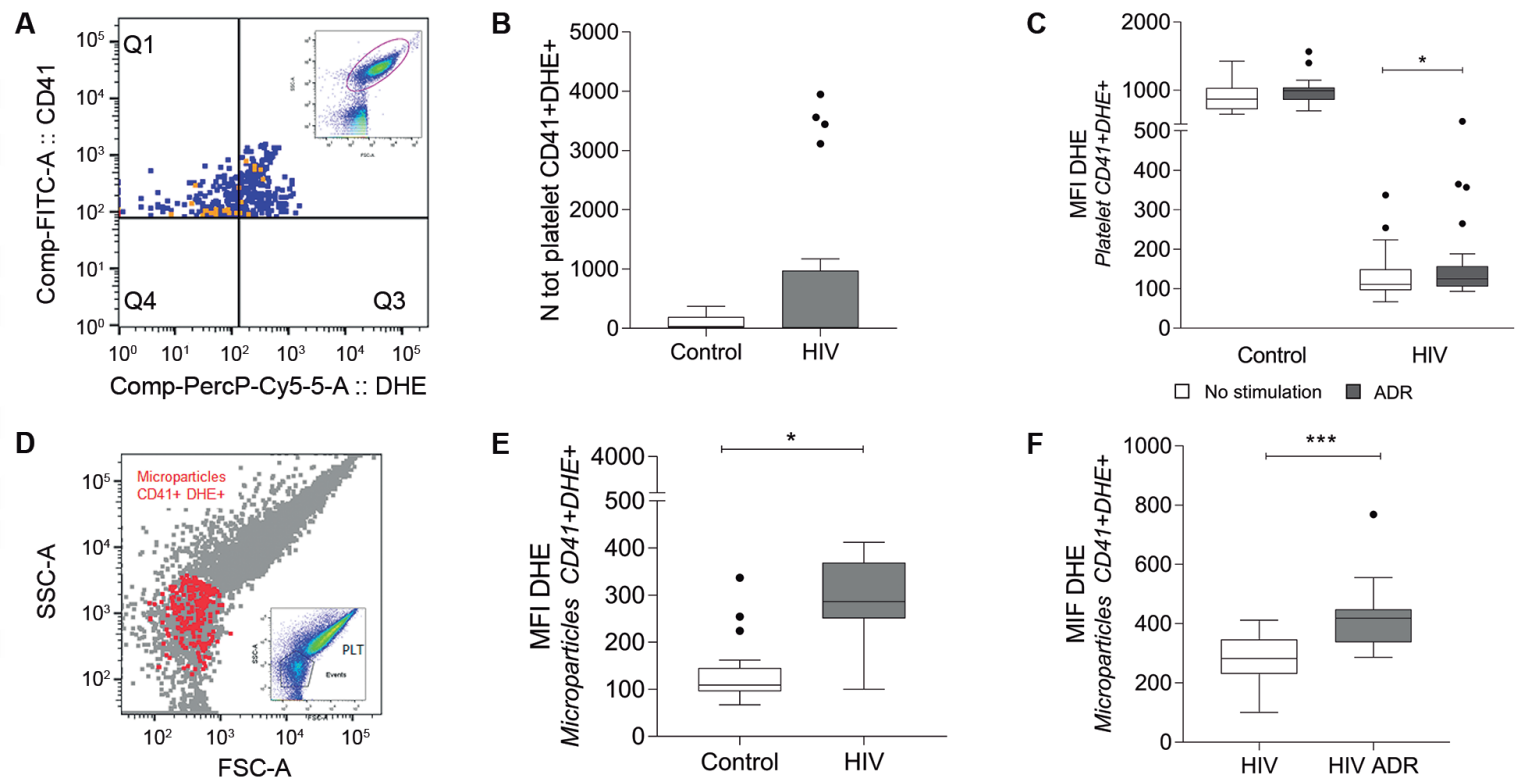
Characterisation of platelets and platelet-derived microparticles by double labeling with anti CD41 and dihydroethidium (DHE). (A) The CD41b and DHE double positive platelets selected from Forward and Size scatter graph (small graph in the upper right corner), distributed in the shape of a comet, which is characteristic of the heterogeneous size distribution (selection). (B) Comparison of the total number of CD41+DHE+ platelets between HIV/AIDS patients and healthy subjects. (C) reactive oxygen species (ROS) levels in platelets before and after stimulation with 200 μg/mL adrenaline determined by the median fluorescence intensity (MFI). Platelets of HIV/AIDS patients increased the level of ROS compared to the control platelets. (D) The CD41b and DHE double positive microplatelets selected from Forward and Size scatter graph. The region below the comet-shaped platelet population were selected for determination of CD41+DHE+ microplatelets (small graph in the lower right corner). The CD41 and DHE double microplatelet populations were defined by lateral and frontal dispersion characteristics and for the expression of double labeling on CD41+ (the FL-1 channel panel) and DHE labeling in FL2 channel panel. Red dot plots indicate CD41+DHE+ microplatelets among events below the comet-shaped platelet population. (E) Comparison of the total number of CD41+DHE+ microplatelets between HIV/AIDS patients and healthy subjects. (F) ROS levels in microplatelets of HIV/AIDS patients before and after adrenaline stimulation with 200 μg/mL of adrenaline for 30 minutes.

It has been suggested that platelet activation generates platelet-derived microparticles (P-MP) in HIV infection.^15^ ^,^ ^16^ ^,^ ^17^ We selected the P-MP for their low lateral and frontal dispersion characteristics and for the expression of CD41+DHE+ below the platelet population, according to other authors (Figure D).^17^ The mean of P-MPs generated in relation to number of CD41+DHE+ platelets was 19.15±157.5. HIV/AIDS patients showed a greater number of P-MPs (Figure E) and responded to stimulus by adrenaline by increasing ROS levels due to the increase of MFI DHE (Figure F). Our clinical interest focused on P-MPs, and a limitation of our study was that it used only anti CD41 and DHE as markers for ROS activity, whereas most eukaryotic cells possess the capacity to release microparticles, as observed in other studies.^18^ An *in vitro* study, greater intracellular ROS production was demonstrated in endothelial cells treated with microparticles from HIV-1 seropositive men, when compared to cells treated with microparticles from healthy individuals.^17^ Such results indicate that this intracellular production of ROS induces endothelial activation, damage and dysfunction, and thus promotes a very common pro-oxidative endothelial phenotype in HIV infection, which is capable of justifying the high risk of developing adverse vascular events during infection.^15^ In addition, it has been reported that microparticles can also induce a pro-atherogenic endothelial cell profile and potentiating apoptosis.^17^ Thus, an intense and persistent activation of platelets/microparticles can contribute to increased vascular risks which underlie HIV-associated endothelial dysfunction with thrombotic events and vascular diseases.^18^ ^,^ ^19^ ^,^ ^20^ Therefore, our findings emphasise that an increased oxidative stress in platelets and P-MPs may increase the risk of RF during HIV-TB coinfection.
